# Cryogenic low-noise amplifiers for measurements with superconducting detectors

**DOI:** 10.3762/bjnano.11.115

**Published:** 2020-09-02

**Authors:** Ilya L Novikov, Boris I Ivanov, Dmitri V Ponomarev, Aleksey G Vostretsov

**Affiliations:** 1 Novosibirsk State Technical University, K.Marx-Av.20, Novosibirsk 630073, Russia

**Keywords:** cryogenic amplifier, cryogenic low-noise amplifier, differential cryogenic amplifier, superconducting circuit readout

## Abstract

We designed, implemented, and characterized differential amplifiers for cryogenic temperatures based on Si bipolar junction transistor technology. The amplifiers show high gain values of more than 60 dB at 300, 77, and 48 K. The minimum voltage noise spectral density was achieved at 77 K and corresponded to 0.33 nV/Hz^0.5^ with a flicker noise of 20 Hz. The maximum voltage gain was 70 dB at 77 K for a frequency range from DC to 17 kHz. We experimentally show that the parallel differential circuit design allows for a reduction of the voltage noise from 0.55 to 0.33 nV/Hz^0.5^ at 77 K.

## Introduction

Currently, superconducting detectors are the most sensitive devices in the electromagnetic field and find wide application in radioastronomy and quantum electronics. Sensors based on superconductors can detect microwaves close to the single-photon limit [1]. Most of such sensors are based on Josephson junctions and superconducting thin films. Experimental studies of such sensors require the design of low-noise cryogenic readout electronics with a direct coupling to the sample. For example, investigations of noise sources in low-temperature tunnel Josephson junctions are still ongoing for high-precision calibration of superconductor technology and for finding new noise sources in Josephson junctions, which lead to high decoherence in superconducting systems [2–3]. The most important part in a measurement readout is a low-noise amplifier. The modern low-temperature low-noise cryogenic amplifiers are widely used for superconducting circuit readout at a temperature of 4 K [4–10]. However, the operating frequency range of such amplifiers starts at 10 kHz or higher. This specific design of cryogenic amplifiers is mainly based on two technological concepts, i.e., high electron mobility transistor (HEMT) technology and SiGe bipolar heterojunction technology (HBT).

Low-frequency amplifiers are usually applied as first stage of SQUID readout electronics [11–12] or as the readout of cryogenic bolometers [13]. In both cases the amplifiers have a working temperature of 300 K. Modern low-frequency and low-noise amplifiers are commonly based on Si bipolar junction transistor (BJT) technology and junction field-effect transistor (JFET) technology. Since external interferences can be compensated by differential input circuits, differential amplifier designs are used for these applications. These amplifiers, including instrumentation amplifiers, are usually built as fully monolithic integrated circuits. Due to the complicated circuit design, their parameters are not stable at temperatures below 240 K and they can not be used at cryogenics. However, it is known that single BJT amplifiers can operate at temperatures down to 77 K [14–15,11]. In [11] the authors use a matched transistor pair to develop a low-noise preamplifier for room-temperature SQUID readout electronics and show the possible application in wide temperature range. They achieved a low voltage noise spectral density of 0.33 nV/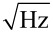 with a 1/*f* flicker noise corner frequency of 0.1 Hz but there is no information about gain and noise performance at 77 K. Moreover, it would be useful to compare room-temperature results of BJT-based differential amplifiers with results obtained at 77 K. Also, modern cryogenic applications are based on dry cryostats instead of coolants. The most common modern dry cryostats, including dilution refrigerators and He-7 sorption refrigerators, have a 50 K temperature stage as uppermost cooling stage where the DC cryogenic amplifier can be mounted.

In this paper, we show the potential applicability of commercially available Si bipolar transistors for cryogenics. Design and preliminary performance of cryogenic low-noise differential amplifiers, based on matched parallel connected pairs of commercially available bipolar junction transistors, at temperatures of 77 and 50 K are presented. This amplifier design is simple to modify, fast to implement, and requires only a proper selection of components. The amplifiers show a high gain value of 70 dB for a frequency range from DC to 17 kHz, a low voltage noise spectral density of 0.33 nV/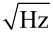 and low flicker noise at 77 K. The total power consumption for the parallel circuit amplifier design corresponds to 180 mW. We experimentally show that the parallel differential circuit design allows for the reduction of the voltage noise from 0.55 to 0.33 nV/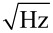 at 77 K. The performance of the amplifier in measurements with superconducting detectors requires improvements, which are currently under investigation.

## Experimental

**Figure 1:**
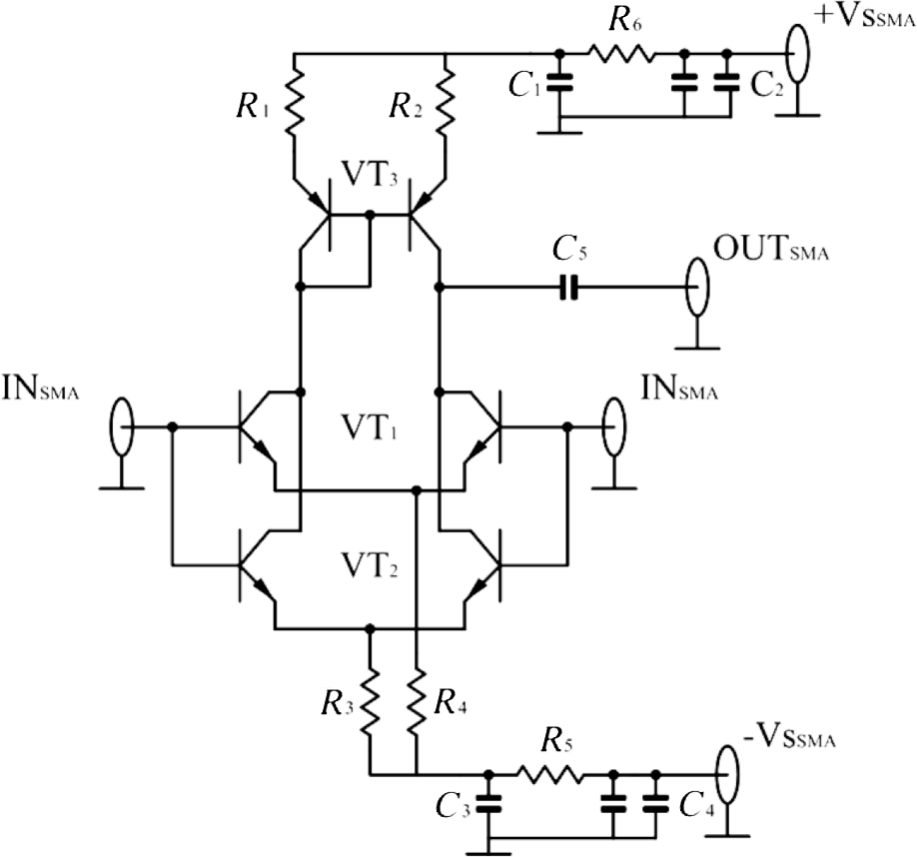
Schematic of the 0–120 kHz cryogenic LNA based on paired SSM2210 transistors. The important component values are: *R*_1_ = *R*_2_ = 100 Ω, *R*_3_ = *R*_4_ = 4.3 kΩ, C_5_ = 470 nF. The capacitors are realized in 0402 package (C0G type) and the resistors are realized as uMELF. The other passive components are used for filtering of the bias lines.

We designed the cryogenic differential amplifier with an active load as a current mirror using negative and positive power supplies. SSM2210 and SSM2220 transistors were used as the main elements. These are n-p-n and p-n-p matched transistor pairs. Each pair is fabricated on one chip. Both type of transistors are used in low-frequency low-noise room-temperature amplifiers for SQUID and bolometer readouts [16–18]. Since the current gain in the bipolar transistors decreases with a lowering of the temperature, the main goal of this circuit design was to achieve a high voltage gain level at cryogenic temperatures of 77 and 48 K. This led us to use transistor bias points at high currents up to 10 mA, which is beyond the optimal working points for minimum voltage and current noise criteria. Moreover, this circuit modifies the differential input to the common mode output, which might be easily connected to any coaxial cable in a cryostat. The first amplifier circuit was designed with one transistor pair (SSM2210) connected to the active load (SSM2220). In order to reduce the Johnson–Nyquist noise we used a second transistor pair of SSM transistors connected in parallel to the input differential circuit as it is shown in Figure 1. This design was used in the second cryogenic differential amplifier. We used a negative voltage supply for biasing the emitter circuits over the resistors *R*_3_ and *R*_4_, which were thermally anchored to the 48 K cooling stage. This way we formed a current source and reduced the noise coming from the room-temperature voltage biasing part. Since the resistors were cooled to 48 K their Johnson–Nyquist noise did not influence the circuit. The resistors were measured at 77 K before and a pair of resistors with low resistance spread was chosen. Each pair of the SSM2210 transistors was measured and the input and output *I*–*V* curves were obtained. Pairs with similar transistor parameters were selected. We used simple RC filters in order to suppress any external interference coming from room-temperature voltage sources. The resistors *R*_1_ and *R*_2_ are the load resistors of the collector circuits, and the capacitor C_5_ is the decoupling output capacitor, which has the unwanted effect of reducing the frequency bandwidth at low frequencies. The resistors used in the cryogenic amplifier are MELF-type resistors and the capacitors have a C0G-type of dielectric. All the components were mounted on the designed layout of the printed circuit board (PCB) from FR4 material. We did not solder the electrodes of the capacitors directly to the PCB in order to reduce the mechanical stress during the cooldown cycles. Instead, we used a small 0.25 mm Cu wire to provide a soldering connection to the PCB pads. The nominals for passive elements are given in the caption of Figure 1. The PCB with the cryogenic amplifier circuit was embedded inside a Cu box with SMA input and output connectors for signal lines. The biasing terminals were set up as feedthrough filters.

## Results and Discussion

**Figure 2:**
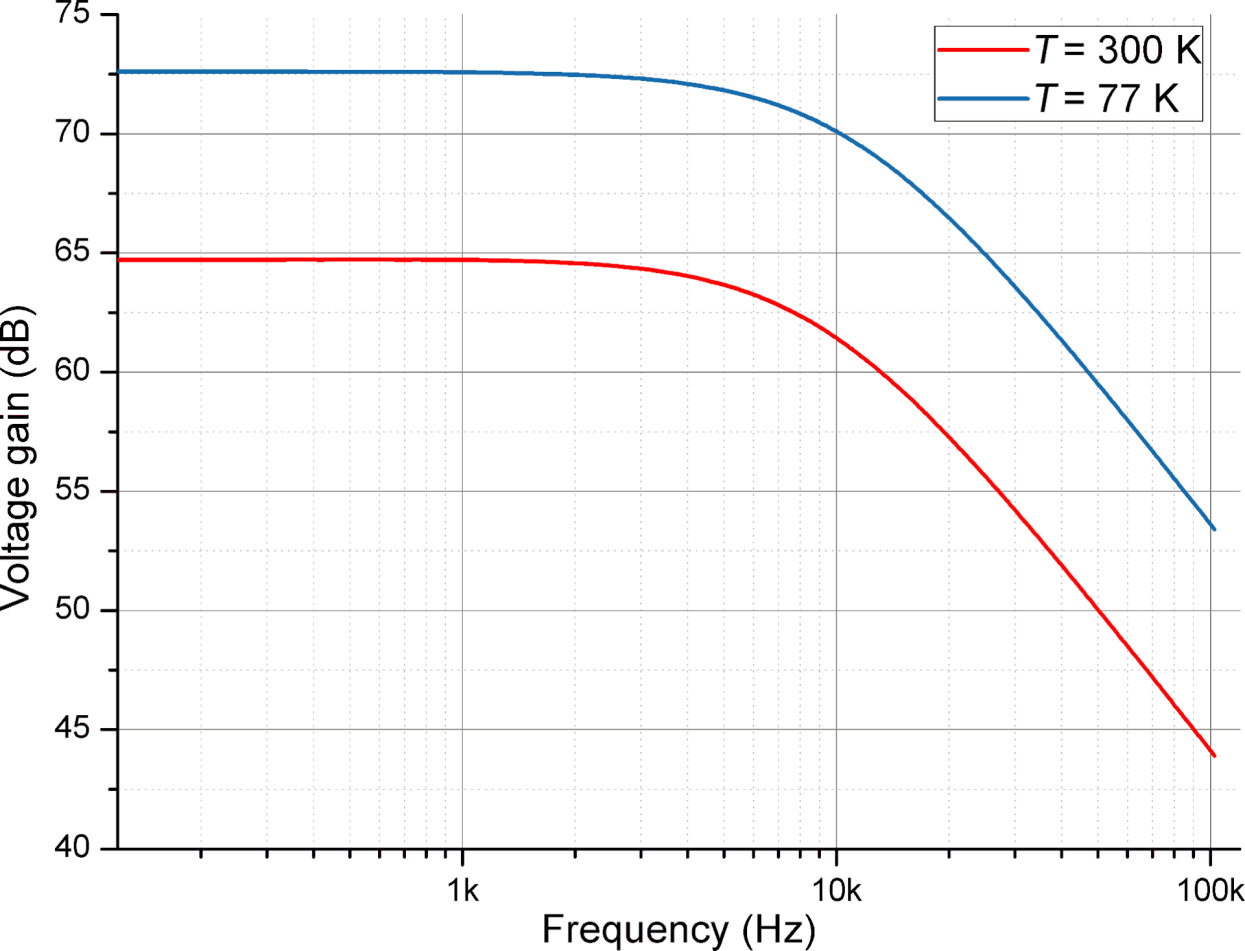
The gain of the cryogenic differential BJT amplifier depending on the frequency at 77 and 300 K.

The main measurement instrument of the setup was a dynamic signal analyzer (SA) SR780 from Stanford Research Systems with a frequency range from DC to 102.4 kHz and imbedded analogue sweeping generator. It was used for gain and noise measurements. We performed measurements of two cryogenic differential amplifiers, i.e., one design with one pair of input transistors and another design with a parallel connection of transistor pairs (see Figure 1). The first design was measured at 300 and 77 K and the second design was characterized at three different temperatures, i.e., 300, 77, and 48 K. The first experimental temperature was room temperature. In order to measure the amplifiers at 77 K we used a liquid nitrogen dewar with good thermal isolation. The amplifier circuit mounted to the Cu case was immersed in a liquid nitrogen dewar. The low-temperature measurements at 48 K were made on the uppermost cooling stage of He-7 refrigerator with a Cryomech pulse tube cryocooler PT-405. Flexible shielded coaxial cables with SMA connectors were used for circuit biasing and for the input and output. We used an additional 40 dB attenuator at the SR780 generator output in order to reduce the voltage amplitude, so that the input voltage signal applied to the amplifiers corresponded to 100 μV. We estimated the dynamic behavior of the amplifiers while applying a sinusoidal analog signal with different amplitudes. The linear voltage range corresponded up to 5 mV at the input. At cryogenic temperatures the amplifiers were measured in two cooldown steps. First, the dependence of the gain on the frequency was obtained and during the next cooldown the voltage noise properties were characterized. The amplifier gain curves measured at two temperatures are presented in Figure 2. The gain of the amplifier corresponded to 73 dB in a frequency range from DC to 11 kHz at 77 K and 65 dB in a frequency range from DC to 9.6 kHz at 300 K.

**Figure 3:**
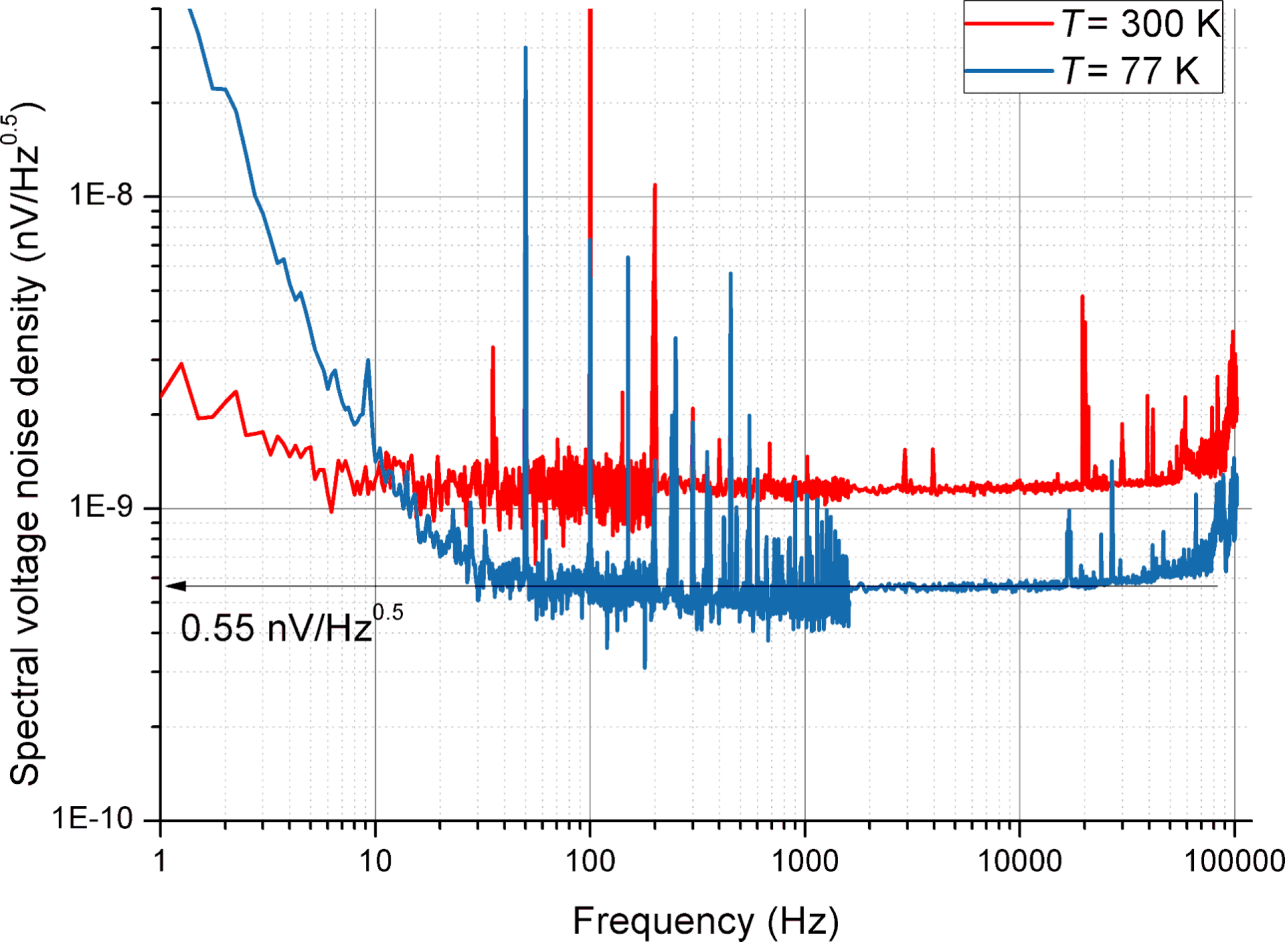
The voltage noise spectral density of the cryogenic differential BJT amplifier as a fucntion of the frequency at 77 and 300 K.

The noise parameters of the amplifiers were measured using a standard measurement procedure. Both inputs of the amplifier were shorted to the ground and the total voltage noise spectral density was measured. The self voltage noise of the SA was previously characterized and it did not exceed 12 nV/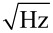. The measured total noise was defined only by the amplifiers since the voltage gain was more than 3000 for both designs. The voltage noise spectral density related to the input as a function of the frequency is presented in Figure 3. The minimum obtained noise was measured at 77 K and corresponded to 0.55 nV/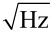. The important parameter for a low-frequency application is the value of the 1/*f* flicker noise frequency. It corresponds to 5 and 20 Hz (Figure 3).

**Figure 4:**
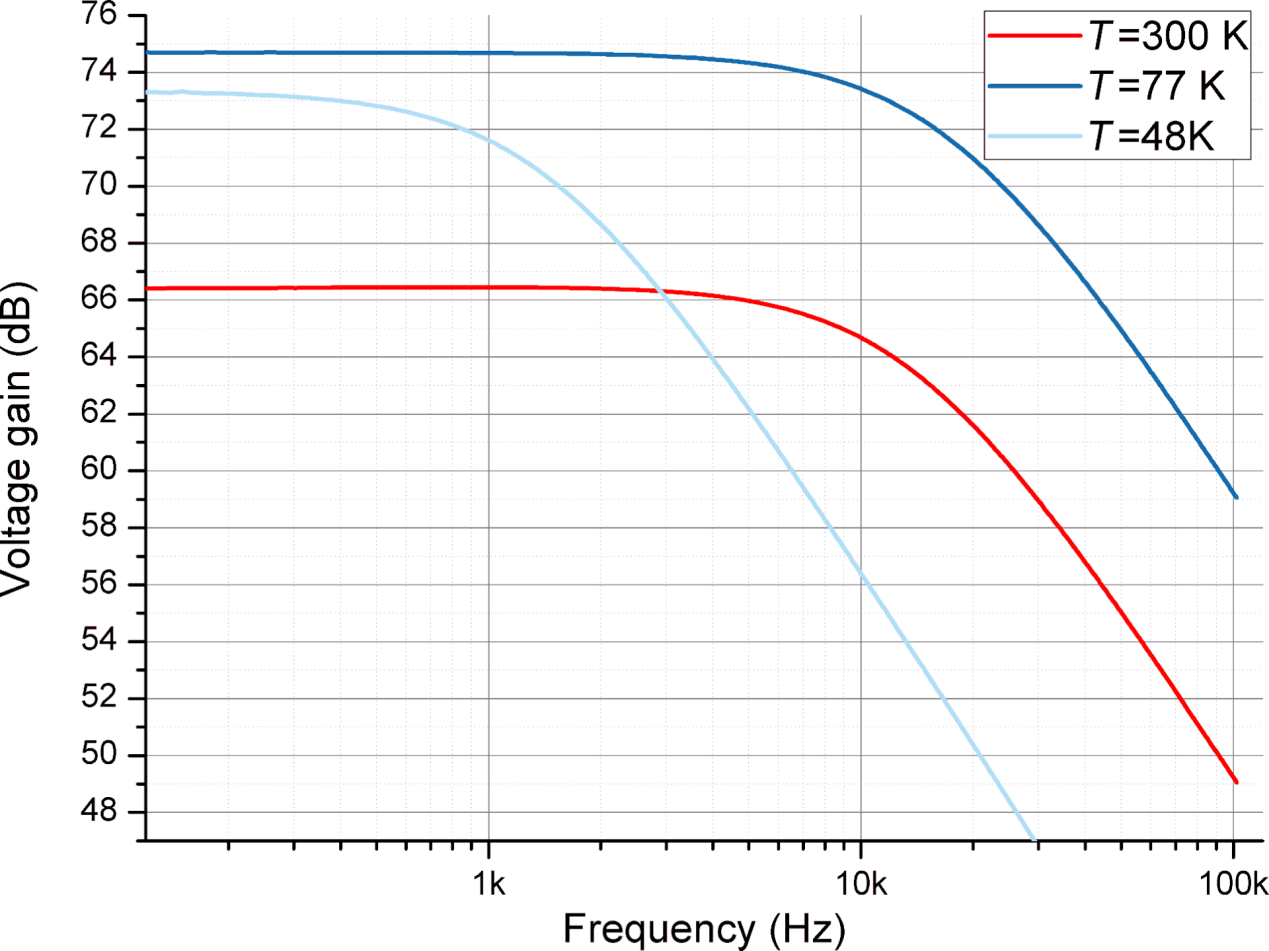
The gain of the cryogenic differential two-stage BJT amplifier as a function of the frequency at three different temperatures.

The second amplifier with a parallel design of SSM2210 transistors (see Figure 1) was conceived to reduce the voltage noise. It was measured at three different temperatures, i.e., 300, 77, and 48 K. In order to measure the amplifier at 48 K we used the uppermost stage of the He cryostat with He-7 sorption unit. The amplifier was fixed to the cooling plate with brass screws to have good thermal anchoring. The signal lines were connected to the cryostat CuBe coaxial cables. The amplifier gain curves measured at three temperatures are presented in Figure 4. The maximum gain of the amplifier was 75 dB, obtained at 77 K. The bandwidth of this amplifier ranged from DC to 17 kHz.

**Figure 5:**
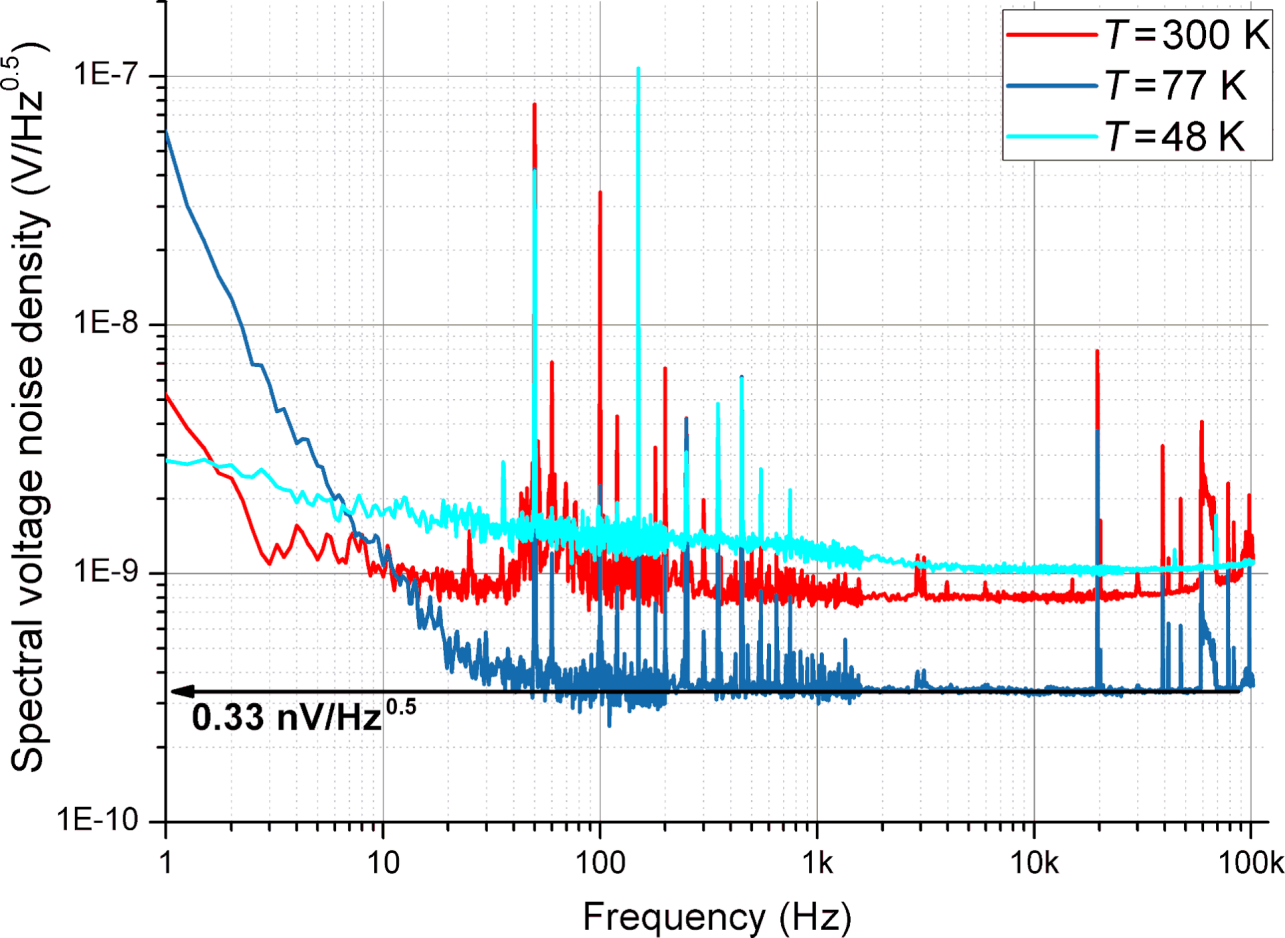
The voltage noise spectral density of the cryogenic differential parallel BJT amplifier as a function of the frequency for three different temperatures. A spurious interference at 50 Hz and its harmonics are visible in the spectrum.

The voltage noise spectral density related to the input as a function of the frequency for three different temperatures is presented in Figure 5. The minimum obtained noise was measured at 77 K and corresponded to 0.33 nV/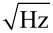. The 1/*f* flicker noise corner frequency corresponded to 5, 20, and about 100 Hz for the different temperatures (Figure 5).

We assume that the high level of 1/*f* noise at 48 K was caused by significant current noise due to a carrier freeze-out effect in the transistor base. Such an effect significantly increases the base current of the transistor to hundreds of milliamperes and increases the spread base resistance at our DC working points. Also, the current gain β was drastically decreased down to approximately five. The characteristics at 48 and 77 K differ, e.g., regarding the amplitude of the 50 Hz interference and its harmonics. This is explained by the different experimental conditions. The 77 K experiments were performed in a liquid nitrogen bath with shielded amplifier case and coaxial lines while the 48 K experiments were performed in the cryostat.

## Conclusion

We have demonstrated differential cryogenic low-frequency low-noise bipolar junction transistor amplifiers. The amplifiers have a high gain value of more than 60 dB, with a voltage noise spectral density of less than 0.55 nV/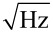 and a low flicker noise of 20 Hz at 77 K. We have shown experimentally that the parallel differential circuit design allows for a reduction of the voltage noise from 0.55 to 0.33 nV/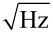. However, the amplifier characteristics were drastically degraded at 50 K. The bandwidth was reduced to 1.5 kHz and the voltage noise spectral density increased to 1 nV/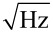. This may limit the usage of the amplifiers for some experimental needs. Generally, the amplifier performance is not sufficient to be a replacement of modern low-frequency amplifiers for SQUID or bolometer readouts. Comparing the performance of this amplifier at 77 K with that of the amplifier from [11] at room temperature we can see worse bandwidth, higher 1/*f* corner frequency and approximately the same voltage noise level. Also, the measurements of amplifier current noise were not carried out because the amplifier was biased by high currents.

However, we want to emphasize that our amplifier design was made to demonstrate the potential applicability of BJ transistors for cryogenic application, especially at 50 K. It was not optimized for SQUID or bolometer readouts. Also the type of transistors were chosen to be n-p-n and p-n-p pairs as in [11]. SSM transistors have the same producer, similar technology, and performance and were used to develop room-temperature SQUID readout electronics [16]. Taking into account a comparison of the performance of the two transistor types SSM and MAT, we are confident that the amplifier performance can be significantly improved. The amplifiers are easy to mount to most of the measurement setups since they have a SMA termination. Unfortunately, modern cryostats for low-temperature experiments do not have a cooling temperature stage corresponding to 77 K. Hence, the designed amplifiers are most applicable for the working temperature of liquid nitrogen, which is the main working temperature for high-temperature superconducting sensors and semiconductor detectors [19–20].
